# Spondylodiscitis in a healthy 12-year-old girl with Extraintestinal pathogenic *Escherichia coli* (ExPEC) bacteraemia

**DOI:** 10.1186/s12879-017-2486-6

**Published:** 2017-05-31

**Authors:** J. Gaschignard, G. Geslain, C. Mallet, M. Lorrot, N. Blot, M. Alison, S. Bonacorsi

**Affiliations:** 1 Service de Pédiatrie Générale, Hôpital Robert-Debré, Paris, France; 2 Université Paris Diderot, Sorbonne Paris Cité, Paris, France; 3 Service de Microbiologie, Hôpital Robert Debré, Paris, France; 4 Service d’Orthopédie pédiatrique, Hôpital Robert Debré, Paris, France; 5 Service de Pédiatrie Générale, Hôpital de Courbevoie-Neuilly, Neuilly-sur-Seine, France; 6 Service de Radiologie, Hôpital Robert Debré, Paris, France

## Abstract

**Background:**

*Escherichia coli (E. coli)* is rarely implicated in bone or joint infections in children.

**Case presentation:**

We discuss the case of a healthy 12-year-old girl with an *E. coli* bacteraemia and a T11-T12 spondylodiscitis revealed by magnetic resonance imaging. The strain harboured serogroup O1:K1 and virulence factors common to highly virulent extra intestinal pathogenic *E. coli* (ExPEC). Immunological work-up was normal.

**Conclusion:**

The identification of *E. coli* in a spondylodiscitis should lead to the search for immunosuppression of the host and virulence factors of the strain, particularly those of ExPEC.

## Background

Bone and joint infections are common in children, and spine is affected in 1–4% of cases [1]. Native vertebral osteomyelitis is often the result of hematogenous seeding of the adjacent disc space from a distant focus, as the disc is avascular. Pyogenic spondylodiscitis usually occur in young children, and the main pathogen is *Staphylococcus aureus* [2]; Gram-negative bacteria are rarely implicated in children as in adults. Among Gram-negative bacteria, *E. coli* is the most frequent in spondylodiscitis in adults [3]; the main risk factors are a pre-existing or synchronous genitourinary tract infection or an intra-abdominal infection [3, 4].

## Case presentation

A 12-year-old caucasian girl presented with a 1-day history of fever (39 °C) with right lumbar pain. She had no medical history and had never travelled outside Western Europe. There was no report of dysuria, frequent daytime urination, abdominal pain or diarrhoea. Physical examination was normal apart from a right lumbar punch. White blood cell (WBC) count was 9400/mm^3^, neutrophils 7600/mm^3^ and CRP 16 mg/L. Blood and urines were collected for culture and ceftriaxone was started for suspicion of pyelonephritis. Urinalysis was negative for leukocyte, nitrite and pyuria. Urinary culture was sterile but one blood culture was positive for an *Escherichia coli* strain susceptible to all antibiotics. Abdominal ultrasound and abdominal tomodensitometry with intravenous injection of an iodised contrast product were normal. The patient was afebrile after 48 h of ceftriaxone. C-Reactive Protein (CRP) culminated at 78 mg/L on day 2 and lowered at 12 mg/L on day 5.

**Fig. 1:**
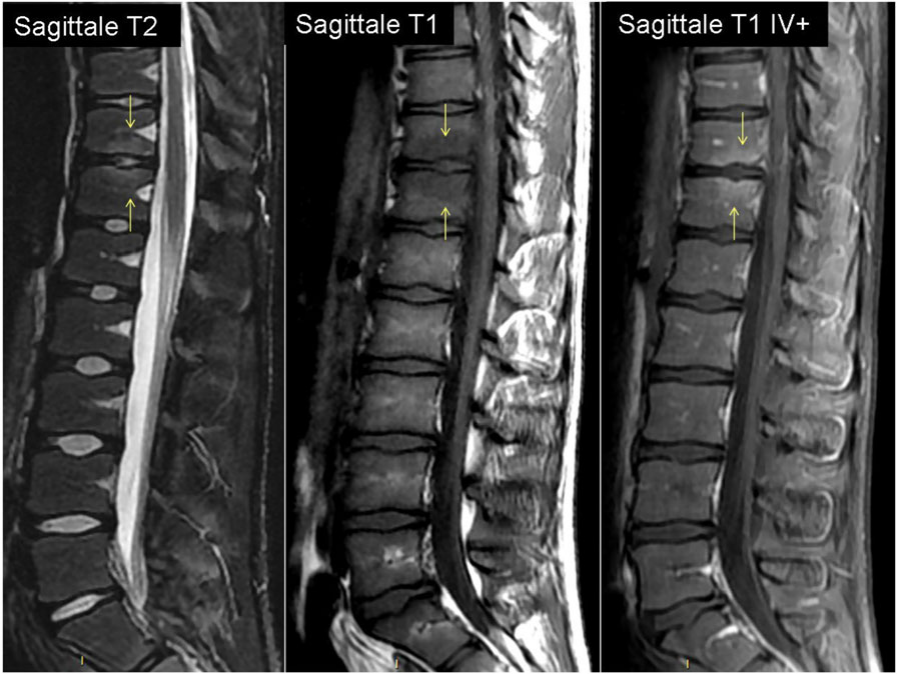
Magnetic resonance imaging of the spine showing a T11-T12 spondylodiscitis. Sagittal T2-weighted, T1-weighted and contrast-enhanced T1-weighted sequences demonstrate mirror oedema (hypersignal T2, hyposignal T1 enhancing after contrast injection) of vertebral adjacent endplates (inferior endplate of T11 and superior endplate of T12) surrounding the intervertebral disk T11-T12, which has decreased T2 signal. There is no soft tissue or epidural extension of the inflammation. With courtesy of Dr. S. Lamer (CH Courbevoie-Neuilly)

However, lumbar pain persisted despite paracetamol and required morphine. A second renal ultrasound was performed and showed no sign of urinary lithiasis. A magnetic resonance imaging of the spine on day 7 revealed a T11-T12 spondylodiscitis without adjacent epidural or soft tissue inflammation (Fig. 1). No bone biopsy was attempted. It would have been considered if the clinical evolution had been unfavourable despite antibiotic treatment. A corset was prescribed and ciprofloxacin was added on day 6 to complete a 14-day course of intravenous antibiotherapy. She had minimal back pain on day 14 and was discharged with oral ciprofloxacin for another 4 weeks. She complained of arthralgia of both wrists on day 35. Physical examination was normal. Ciprofloxacin was stopped on day 42 and arthralgia resolved spontaneously.

Polymerase chain reactions (PCR) were performed to analyze *E. coli* strain, as described previously [5–7]. It harbored serogroup O1:K1 and belonged to the major phylogenetic group B2_1_. Among 23 genetic determinants encoding virulence factors (VFs) investigated [5–7]. 14 were present, including genes coding for siderophores (yersiniabactin - *fyuA*, aerobactin - *iucC*, *sit* system - *sitA*, salmochelin – *iroN*), adhesins P *fimbriae* (*papGII but not papGIII*), *pilin* (*papC*), vacuolating toxin (*vat*) and cytolethal distending toxin (*cdt*). Moreover, strain had other genetic determinants: colicin V (*cvaA*), colicin Ia (*cia*), a pore-forming toxin gene that increases serum survival (*iss)*, a type I secretion system operon (*ets*), a putative outer membrane protease (*ompT*) and a hemolysin (*hlyF*) which combined with the aerobactin and salmochelin genes are characteristic of a conserved virulence plasmidic region identified in the highly virulent extra intestinal pathogenic *E. coli* strain S88 [6]. Finally, PCR were negative for other adhesin/invasin genes (*sfa/foc* and *ibeA*), alpha-hemolysin (*hlyA*), cytotoxic necrotizing factor (*nf1*), secreted autotransporter toxin (*sat*) and colibactin (*clbB*).

Immunological explorations including WBC count and smear, the determination of plasma IgA, G, M and E and IgG subclasses levels and classic complement pathway analysis were normal. The absence of previous unusual infection in our patient didn’t lead us further explores her immunity to rule out an immunodeficiency.

## Discussion

Here, our patient had no diarrhoea, and urine culture and imaging ruled out a genitourinary tract or intestinal infection. There was no history of trauma or injury associated with the spine. We speculated the spine was involved because of the good vascularisation of the disk in children. Moreover, she had no underlying condition and immunological work-up didn’t favour a primary immunodeficiency. The characterization of the strain of *E. coli* involved in our case, particularly its virulence factors, was therefore instructive.

Extraintestinal pathogenic *Escherichia coli* (ExPEC) bloodstream infections represent a growing public health concern [8, 9]. ExPEC strains have acquired genes encoding virulence factors that allow them to cause infections outside the gastrointestinal tract. The B2 phylogenetic group is the main one implicated in various extra intestinal infections [10] and in 63% of *E. coli* bacteraemia in French children [11]. This proportion is similar whether the identified portal of entry is the urinary tract or the digestive tract. The most frequent extra-intestinal virulence genes identified in this nation-wide study were *fyuA*, *irp2*, *iraT*, *ompT*, *iucC*, *iron*, *papC* and *papGII*. Our strain belonged to the B2_1_ subgroup, one of the most virulent subgroup, and harbored 6 of these 8 virulence factors, namely *fyuA*, *ompT*, *iucC*, *iron*, *papC* and *papGII*: those could explain the predisposition of this strain to be bacteraemic. Virulence factors of our strain included adhesins (e.g. P fimbriae), factors to avoid or subvert host defense systems (e.g. capsule K1) and nutrient acquisition factors (e.g. siderophores). However, to our knowledge, no publication has established a link between some virulence factors and bone/joint infections.

Recently, Lemaitre et al. and others showed that the conserved virulence plasmidic region identified in our strain contributed to the high level of bacteremia in neonates [6–12]. The serotype O1:K1 could also contribute to the virulence of our strain. Beside K1 capsule antigen that provides a survival advantage in serum [13], O1-antigen serotype might also confer protection against complement killing [14]. However, our strain didn’t have genes *hlyA* (alpha-hemolysin) nor *cnf1* (cytotoxic necrotic factor), two virulence factors identified as highly cytotoxic toward human osteoblastic cells in vitro [15].

Concerning the antibiotic management of our patient, the French Society for Infectious Diseases recommends intravenous association of a 3rd generation cephalosporin and a fluoroquinolon to treat spondylodiscitis caused by *E. coli*. The duration of IV therapy or combination therapy is not precised in the recommendations. Given the good clinical evolution and the excellent oral biodisponibility of ciprofloxacin, intravenous and association were stopped on day 14 for oral ciprofloxacin alone.

## Conclusions

*Escherichia coli* is an uncommon cause of spondylodiscitis, particularly in children. The strain harbored serogroup O1:K1 and virulence factors common to highly virulent extra intestinal pathogenic *E. coli* that could explain this rare clinical presentation. The identification of *E. coli* in a spondylodiscitis should lead to the search for immunosuppression of the host and virulence factors of the strain, particularly those of ExPEC.

## Abbreviations

CRP: C-reactive protein
*E. coli*: *Escherichia coli*
Expec: Extra intestinal pathogenic *Escherichia coli*
Ig: Immunoglobulin
IV: Intravenous
PCR: Polymerase chain reaction
WBC: White blood cells
